# T55. DRIVING ABILITIES IN CLINICALLY STABLE OUTPATIENTS WITH SCHIZOPHRENIA

**DOI:** 10.1093/schbul/sby016.331

**Published:** 2018-04-01

**Authors:** Falko Biedermann, Alex Hofer, Theresia Pichker, Sylvia Pardeller, Georg Kemmler, Bernhard Holzner, Ilse Kurzthaler

**Affiliations:** Medical University Innsbruck

## Abstract

**Background:**

According to the UN convention of human rights, individual mobility is an important aspect for people suffering from chronic disease. Recent studies have shown that 30% of patients suffering from schizophrenia have a driving license for motorized vehicles, however, studies on driving abilities among this patient group are scarce. Accordingly, the current study investigates the parameters, which are relevant in this regard.

**Methods:**

In this naturalistic study, stable patients, diagnosed with schizophrenia according to ICD-10, between 18 and 60 years of age, are recruited on an outpatient basis. They have to be clinically stable without hospitalization for at least 6 months and have to be on the same medication for at least 6 months. Psychopathology and extrapyramidal motor symptoms (EPS) are assessed by means of the Positive and Negative Syndrome Scale (PANSS) and the Modified Simpson-Angus Scale (MSAS), respectively. Driving abilities are investigated by means of a computerized test battery of the Wiener Testsystem, measuring visual perception, reactivity and stress tolerance, concentration, vigilance, and motor coordination.

**Results:**

So far, 42 outpatients suffering from schizophrenia, with a mean age of 42.7 ± 8.9 years and a mean duration of illness of 11.2 ± 5.5 years, have been included into the study. 52 % were male and the mean education was 14.4 ± 4.0 years. The mean PANSS total score was 56.3 ± 20.3 (positive symptoms: 12.9 ± 5.4, negative symptoms: 13.6 ± 5.5, general symptoms: 29.7 ± 13.6). All patients were treated with second generation antipsychotics, and only one had a combination therapy with an additional first generation antipsychotic. We found significant positive correlations between driving abilities and both years of education and EPS, whereas residual symptoms (PANSS) were not associated with driving abilities.

**Discussion:**

The relationship between EPS and driving abilities was not surprising, since motor flexibility might be seen as basic requirement in traffic situations. The missing correlation between residual symptomatology and driving abilities, on the other hand, may be explained by very low mean PANSS scores and the small range of scores in our sample. To summarize, these data suggest that in clinically stable outpatients suffering from schizophrenia driving abilities are primarily influenced by EPS rather than by residual symptomatology. Altogether, further studies are needed with a larger sample size.

